# Supplementary material for: Invasive Bacterial Infections of the Musculoskeletal and Central Nervous System during Pig Rearing: Detection Frequencies of Different Pathogens and Specific Streptococcus suis Genotypes
Source: Vet Sci. 2024 Jan 2;11(1):17. doi: 10.3390/vetsci11010017 (PMC10820919; doi:10.3390/vetsci11010017)
Supplement: Supplementary file 1 [file vetsci-11-00017-s001.zip › vetsci-2734219-supplementary.pdf]

Supplementary materials

Table S1. Results of *S. suis cps* typing with MP PCR.

sites	number of isolates (n=26)	*cps* type (*)
meningeal swab	2	8, nt
lung	4	8 (2),13, nt
kidney	4	23,28, nt (2)
Lnn. cervicales superficiales dexter	1	nt
Lnn. bifurcationis medii	2	24, nt
Lnn. gastrici	5	8, 11, 27, nt (2)
Lnn. jejunales	1	31
Lnn. colici	2	8 (2)
Lnn. iliaci mediales dexter	3	28, nt (2)
further site[1]	2	3, 8

* Number of isolates with the mentioned capsule type, if >1. nt: non-typable. [1]isolates from heart valve (*cps* 3) and abscess rip (*cps* 8).

Table S2. Results of the mismatch amplification mutation assay (MAMA) for distinguishing *cps*2 and *cps*1/2.

site	number of isolates (n=233)	*cps* type (*)
meningeal swab	15	1/2 (2), 2 (12), nd
lung	2	1/2 (2)
kidney	2	1/2, 2
Lnn. cervicales superficiales dexter	1	2
Lnn. gastrici	1	1/2
Lnn. inguinales superficiales dexter	1	2
further site[1]	1	1/2

* Number of isolates with the mentioned capsule type, if n>1. nd: not distinguished (isolate can be *cps* 2 or *cps* 1/2). [1]inner ear swab.

Table S3. Number of pathotypes of *S. suis* isolates.

pathotype	Number of all detected *S. suis* isolates	Number of *S. suis* isolates after correction of multiple counting per herd
*cps*1 *mrp+ sly+ epf+*	19	18
*cps*1 *mrp- sly+ epf-*	2	2
*cps*1/2 *mrp+ sly+ epf+*	31	21
*cps*1/2 *mrp+ sly- epf-*	9	7
*cps*2 *mrp+ sly+ epf+*	134	75
*cps*2 *mrp+ sly- epf-*	9	9
*cps*2 *mrp- sly+ epf+*	12	12
*cps*2 or 1/2 *mrp+ sly- epf-*	10	10
*cps*2 or 1/2 *mrp- sly+ epf-*	1	1
*cps*3 *mrp+ sly- epf-*	4	4
*cps*4 *mrp+ sly+ epf-*	25	25
*cps*4 *mrp- sly+ epf-*	3	3
*cps*7 *mrp+ sly- epf-*	32	27

*cps*8 *mrp+ sly+ epf-*	1	1
*cps*8 *mrp- sly+ epf+*	0	0
*cps*8 *mrp- sly+ epf-*	8	7
*cps*9 *mrp+ sly+ epf-*	181	126
*cps*9 *mrp- sly+ epf+*	6	5
*cps*9 *mrp- sly+ epf-*	12	12
*cps*9 *mrp- sly- epf-*	4	4
*cps*11 *mrp+ sly+ epf-*	1	1
*cps*13 *mrp- sly- epf-*	1	1
*cps*14 *mrp+ sly+ epf+*	1	1
*cps*23 *mrp+ sly+ epf-*	1	1
*cps*24 *mrp- sly- epf-*	1	1
*cps*27 *mrp- sly- epf-*	2	2
*cps*28 *mrp- sly+ epf-*	2	2
*cps*31 *mrp- sly- epf-*	1	1
nt *mrp+ sly+ epf-*	1	1
nt *mrp+ sly- epf-*	3	3
nt *mrp- sly+ epf+*	2	2
nt *mrp- sly+ epf-*	3	3
nt *mrp- sly- epf-*	7	7
**total**	**529**	**395**

nt: non-typable. Gene detected (+). Gene not detected (-)

Table S4. Number of *cps* types of *S. suis* isolates after correction of multiple counting per herd.

cps type	pathotype	joint swab	meningeal swab	lung	kidney	*Ln. cerv. supf.*	*Ln. bif. m.*	*Ln. gastricus*	*Ln. jejunalis*	*Ln. ileocolicus*	*Ln. colicus*	*Ln. iliacus m.*	*Ln.* ing. supf.	further site[1]	total
**1**	total	5	2	1	2	1	1	1	1	1	0	0	0	5	20
	mrp+ sly+ epf+	4	1	1	2	1	1	1	1	1	0	0	0	5	18
	mrp- sly+ epf -	1	1	0	0	0	0	0	0	0	0	0	0	0	2
**1/2**	total	0	2	4	3	2	3	3	2	2	1	2	2	2	28
	mrp+ sly+ epf+	0	2	2	2	2	2	2	2	2	1	2	2	0	21
	mrp+ sly- epf -	0	0	2	1	0	1	1	0	0	0	0	0	2	7
**2**	total	6	12	7	11	11	6	5	5	5	5	7	7	9	96
	mrp+ sly+ epf+	5	10	5	7	9	4	5	4	4	4	5	6	7	75
	mrp+ sly- epf -	0	0	2	2	1	0	0	0	1	0	1	0	2	9
	mrp- sly+ epf+	1	2	0	2	1	2	0	1	0	1	1	1	0	12
**2 or 1/2**	total	1	1	1	1	1	1	1	0	2	1	0	1	0	11
	mrp+ sly- epf-	1	1	1	1	1	1	1	0	1	1	0	1	0	10
	mrp- sly+ epf+	0	0	0	0	0	0	0	0	1	0	0	0	0	1
**3**	*mrp+ sly- epf-*	0	0	0	0	0	1	1	1	0	0	0	0	1	4
**4**	total	3	2	5	3	1	1	3	1	2	3	1	2	1	28
	mrp+ sly+ epf-	3	2	3	3	1	1	2	1	2	3	1	2	1	25
	mrp- sly+ epf-	0	0	2	0	0	0	1	0	0	0	0	0	0	3
**7**	*mrp+ sly- epf-*	2	2	5	3	2	1	1	2	2	1	2	2	2	27
**9**	total	12	18	11	15	12	10	11	11	9	8	10	11	9	147
	mrp+ sly+ epf-	10	15	8	12	11	9	10	9	8	7	10	9	8	126
	mrp- sly+ epf+	0	2	1	1	0	0	0	1	0	0	0	0	0	5
	mrp- sly+ epf-	1	1	2	1	1	0	1	1	1	1	0	1	1	12
	mrp- sly- epf-	1	0	0	1	0	1	0	0	0	0	0	1	0	4

***cps* type**	**pathotype**	**joint swab**	**meningeal swab**	**lung**	**kidney**	***Ln. cerv. supf.***	***Ln. bif. m.***	***Ln. gastricus***	***Ln. jejunalis***	***Ln. ileocolicus***	***Ln. colicus***	***Ln. iliacus m.***	***Ln.* ing. supf.**	**further site[1]**	**total**
**11**	*mrp+ sly+ epf-*	0	0	0	0	0	0	1	0	0	0	0	0	0	1
**13**	*mrp- sly- epf-*	0	0	1	0	0	0	0	0	0	0	0	0	0	1
**14**	*mrp+ sly+ epf+*	1	0	0	0	0	0	0	0	0	0	0	0	0	1
**23**	*mrp+ sly+ ep -*	0	0	0	1	0	0	0	0	0	0	0	0	0	1
**24**	*mrp- sly- epf-*	0	0	0	0	0	1	0	0	0	0	0	0	0	1
**27**	*mrp- sly- epf-*	0	0	0	0	0	0	1	1	0	0	0	0	0	2
**28**	*mrp- sly+ epf-*	0	0	0	1	0	0	0	0	0	0	1	0	0	2
**31**	*mrp- sly- epf-*	0	0	0	0	0	0	0	1	0	0	0	0	0	1
**nt**	total	0	1	2	2	1	1	4	2	0	0	3	0	0	16
	mrp+ sly+ epf-	0	0	0	0	0	0	1	0	0	0	0	0	0	1
	mrp+ sly- epf-	0	0	0	1	0	0	1	0	0	0	1	0	0	3
	mrp- sly+ epf+	0	1	1	0	0	0	0	0	0	0	0	0	0	2
	mrp- sly+ epf-	0	0	1	0	0	0	1	1	0	0	0	0	0	3
	mrp- sly- epf-	0	0	0	1	1	1	1	1	0	0	2	0	0	7
**total**		**30**	**41**	**39**	**42**	**31**	**26**	**34**	**27**	**23**	**21**	**26**	**25**	**30**	**395**

Gene detected (+). Gene not detected (-).[1]Serosal swab (n=9), bursal swab (n=7), heart valve (n=5), spleen (n=3), and another site (n=6).

Table S5. Number of genotypes of *S. suis* isolates (n=529) divided by age category(*S. suis* isolates from all sites examined).

genotype	age category (number of examined animals)			
	**suckling piglet (n=16)**	**weaning piglet (n=93)**	**fattening pig (n=15)**	**total (n=124)**
*cps*1 *mrp*+ *sly*+ *epf*+	5	14	0	19
*cps*1 *mrp* - *sly*+ *epf* -	2	0	0	2
*cps*1/2 *mrp*+ *sly*+ *epf*+	0	31	0	31
*cps*1/2 *mrp*+ *sly* - *epf* -	2	2	5	9
*cps*2 *mrp*+ *sly*+ *epf*+	0	134	0	134
*cps*2 *mrp*+ *sly* - *epf* -	0	9	0	9
*cps*2 *mrp* - *sly*+ *epf*+	0	12	0	12
*cps*2 or 1/2 *mrp*+ *sly* - *epf* -	0	10	0	10
*cps*2 or 1/2 *mrp* - *sly*+ *epf* -	0	1	0	1
*cps*3 *mrp*+ *sly* - *epf* -	0	0	4	4
*cps*4 *mrp*+ *sly*+ *epf* -	3	21	1	25
*cps*4 *mrp* - *sly*+ *epf* -	0	3	0	3
*cps*7 *mrp*+ *sly* - *epf* -	17	12	3	32
*cps*8 *mrp*+ *sly*+ *epf* -	0	1	0	1
*cps*8 *mrp* - *sly*+ *epf* -	3	3	2	8
*cps*9 *mrp*+ *sly*+ *epf* -	0	173	8	181
*cps*9 *mrp* - *sly*+ *epf*+	0	6	0	6
*cps*9 *mrp* - *sly*+ *epf* -	0	11	1	12
*cps*9 *mrp* - *sly* - *epf* -	0	3	1	4
*cps*11 *mrp*+ *sly*+ *epf* -	0	0	1	1
*cps*13 *mrp* - *sly* - *epf* -	0	1	0	1
*cps*14 *mrp*+ *sly*+ *epf*+	0	1	0	1
*cps*23 *mrp*+ *sly*+ *epf* -	1	0	0	1
*cps*24 *mrp* - *sly* - *epf* -	0	1	0	1
*cps*27 *mrp* - *sly* - *epf* -	0	2	0	2
*cps*28 *mrp* - *sly*+ *epf* -	0	2	0	2
*cps*31 *mrp* - *sly* - *epf* -	0	1	0	1
nt *mrp*+ *sly*+ *epf* -	0	1	0	1
nt *mrp*+ *sly* - *epf* -	0	0	3	3
nt *mrp* - *sly*+ *epf*+	2	0	0	2
nt *mrp* - *sly*+ *epf* -	0	3	0	3
nt *mrp* - *sly* - *epf* -	0	5	2	7
**total**	**35**	**463**	**31**	**529**

nt: non-typable. Gene detected (+). Gene not detected (-).
